# Dental Exhibitions

**Published:** 1854-01

**Authors:** 


*Dental Exhibitions.* By a Dental Surgeon.

Although not accustomed to writing for periodicals, still I consider myself as one of the "profession;" and as every one who loves his profession should look to its public as well as private welfare, I consider myself bound, when I see a call for it, to stand forth in defence of any theory or practice which, although crushed down by others, may be of use to the profession.

I think that no subject is fully settled as a fact until both sides are heard; therefore my object at this time is to review the remarks of your Illinois correspondent, in the Register for October.

In the first place, your correspondent strikes a blow at the periodicals, for publishing what he terms the "conflicting throws" of the zealous members of the profession, as it appears, for this reason—because those who have not ambition and talents to be leaders must be content to follow in the rear.

It is true all who see fit to rank themselves as dentists have not ambition to learn anything new by their own exertion and experiments. Then why should they wish to "gag" those who have an ambition to excel, and are willing to throw before an enlightened public the result of their experiments.

All can not avail themselves of the advantages offered by dental colleges, or even instructions of first-rate operators in private practice. How, then, I would ask, are they of the "backwoods" ever to know of what is going on in the profession, or to be posted up in the improvements of "this age of steam?"

Thanks to the genius of American liberty, "Young America" is in the field—his watchword "onward," the countersign "improvement." And most nobly is he fighting the battles of dental science. The victory is certain, and he heralds his triumph through that most perfect of all channels—the public press. Even while we are now writing, "Young America" is telling of the victories gained by American dentists, as proved at the World's Industrial Exhibition.

He is suppressing "quackery," in all its forms. A few years ago, nearly every dentist had his specifics, infallible remedies, and only known manner of performing certain operations. "Young America" has exposed them all to the world, and scattered the contents of "Pandoras box" to the four winds of heaven. And they of the "backwoods," if they will put themselves to the trouble to read the public prints, can claim their share of the plunder.

"Young America" has robbed us of our "secrets," by which we once gained our practice and living, and stripped dentistry of that mystery which once gave its votaries their importance.

Now I would ask in what way are we to get any recompense for our trouble in experimenting to improve dental science? Your correspondent deprives us of gaining reputation by publishing our "new theory" in the periodicals; he hisses our "flaming advertisements," and he calls it unprofessional to exhibit our work—the work itself, stripped of all theory—at our public exhibitions, or even to have a case of artificial teeth to show our patients in our own private office. How, then, I would again ask, are we to be paid for "our superior operations," or the public to know that we can do superior work? Shall we tell it to the unambitious dentist, and he to our patrons? Will he do it? Will he bring us more custom or fame? Will he raise us from the common herd, like himself, to the station which our talents demand? Can we expect justice or honor from the man who would deprive us of the privilege of publishing to the profession and world our improvements, or exhibiting our handiwork?

It is but a few years since Dr. Solyman Brown, of New York, was reviled by members of the profession, for publishing to the world his papers on mechanical dentistry. They told him that, by publishing the "secrets of dentistry," he was going to ruin the operators. For, say they, then every quack will know as much as we do, and our business be entirely ruined. A good philosophy, truly; but did Dr. Brown injure the profession by publishing his papers? Or is the public at large injured by it? On the contrary, is not the public benefitted largely, and are not all true men of the profession showing their thanks and gratitude to him for coming out boldly, and manfully breaking down that system which was contracting the power of dental science and retarding the progress of improvement.

From him dates a new era in dentistry. He showed to the world that the man who could perform the best operation was worthy of respect and support, rather than he who claimed public confidence by having the largest amount of "secrets." And the facts of artistic skill were to be the criterion for judgment, rather than the mere say-so of the operator.

But I will again return to a review of the work before me. An objection urged against exhibiting our work at public fairs is, that the work which is beautiful and highly finished, and draws the first premium, may have been made by a jeweler, or some one who never saw the adaptation of the job to the mouth.

Now, Mr. Editor, I would ask you if that objection is a good one? Is it necessary that all the work which we give our patients should be done by our own hands? How many of us have workmen, who do all of our mechanical work, who never see any of it adapted? What does it matter, whether it is done by a man called a jeweler, or a dental-plate workman. If we had to do all our plate work with our own hands, I fear few of us would ever accomplish as much as we now do.

What if Jones White & Co. had been refused the premium on their artificial teeth at the World's Fair, because most of them were made by hired workmen; would we not have condemned such a decision? For myself, I would consider such a course as reflecting dishonor on any set of judges.

The man who presents his work before the public for inspection is alone responsible for it. He puts his reputation at stake by exhibiting his work; and if he can not perform as good operations for his patients as he exhibits before the public, his patrons will soon find him out, and his reputation will come down to the standard of his operations. The public are not long deceived by a false show. They are learning how to prove even a dentist. His beautifully gilded sign and ornamental door-plate are not now sufficient guarantees to the public of his abilities in his profession.

For my own part, I consider a *good operation* in dentistry the best advertisement we can throw out to solicit custom. I think it both right and proper for a dentist to advertise, as much so as for a physician or a merchant. We are dependent on the public for custom and support, and why is it wrong for us to solicit their patronage, by showing them what we can do, and the kind of work we are capable of giving them in return for their money?

Your Illinois correspondent says he would not object to exhibiting our work, if he could show it in the mouth of the patient.

That would be nice, truly, but so long as our patients object to such a course, we must, if at all, show it out of the mouth. If a man can show me a good job out of the mouth, I think the inference strong that he can also adapt it to the mouth; at least it is not for me to doubt, until I see proof to the contrary.

The American people have been humbugged so many times that they prefer to see a sample before they buy. They prefer their own eyes in judging, rather than the say-so of any one. Custom has made this rule, that if the articles purchased are not equal to the samples shown, we are not obliged to take them at any price; and I consider a job of artificial teeth as subject to the same rules in trade as any other goods purchased.

Many persons never see a job of artificial teeth until they have occasion to have one themselves. They wish to make many inquiries about the way artificial teeth are made and sustained in the mouth; and if the dentist has a few pieces of work in his office, he can exhibit them to his patient, and illustrate to them how they are adapted to the mouth, also show them how much material it takes to perform a good operation, and why it is we have to charge so high a price for so small a piece of work.

But few persons, unless they have had dental operations before, are aware how high dental charges are, or how much valuable material is necessary to perform the operation.

If all dentists were honest men, and would perform all they promise, and all those who wish for operations had perfect confidence in the honesty and ability of the dentist, then the case would be somewhat different. But, unfortunately for dental science, the "quack" is abroad, and by his great pretensions is drawing patronage which ought to be given to truly scientific and honest operators.

But how is the honest operator ever to get into practice, if he is not allowed to show his work? How are the public ever to know that he can do good work! His own word is not always sufficient, for he may be a stranger.

Does your Illinois correspondent answer that he must remain in a place until he is known to be of good moral character, and then, if he is worthy, he can get custom?

He may be short of means, and before this could be done he would starve; whilə the man who can exhibit a good piece of dental work, even if a stranger, will be doing a fine and lucrative business, and securing a good custom and reputation.

Our patrons are more willing to pay for a good job of work than for the honesty exhibited in doing it. I would not by any means undervalue honesty in a dental operation—far from it—for I think that no one can long succeed in dentistry who is not honest. But I consider it honest in every way to exhibit our work, and show to our friends and the public how and where we have improved on the operations of our ancestors; and if a spittoon or operating chair, which are simply conveniences for the *dentist*, are worthy of commendation, how much more ought the job of work, which is for the *patient's* use, to be proved and commended, and the operator to receive the praise due for his industry and experiments.

Let those who are now so vehement in crying "humbug," bring before an enlightened public their own works, which they say "are beautifying the persons, mellowing the voices, and performing perfectly for gastric elaboration the aliment of scores or hundreds of individuals," let *them*, I say, bring that work before the public, and submit it to a rigid examination, and many, if not all of them would return without receiving a premium on the work, or an encomium on the operator. Instead of hearing the joyful words of "well done, thou good and faithful servant," they would return to their workshops with the withering sentence on their heads, of "depart, ye lazy and worthless pretenders, I know you not."

And now, my brother operators, I challenge you to meet me in the field of dental science. Dentistry is yet in its infancy; fresh laurels are yet to be won, and to be won only by those who fight manfully in the cause. Oppose "quackery" in all its forms, and strike down all who are clogging the wheels of dental improvement. Hold up the secret pretender to public view, and expose his false system to ridicule. Throw your improvements before an intelligent public, and they will reward your industry. Publish the result of your experiments, and let others judge whether they are beneficial and worthy of praise. Support our dental periodicals not only with your money and influeuce, but with your pen. Contribute your mite toward elevating the standard of dental excellence and usefulness. If you have a light, even a dim one, elevate it, so that it may help to enlighten the profession and benefit community. Stand aloof from those who would stop improvement, and willingly plod along in the same old beaten track which their fathers trod.

Lend your influence toward supporting dental colleges and public instruction, and, instead of lowering the standard of dental qualifications, place it still higher, and raise the profession to that honorable station which its importance to the community demands. Be not content to arrive at the place where our ancestors stood, but press onward until the glorious prize of excellence is gained. And when we have passed off from this stage of action, and others have taken our place, may they have cause to eulogize our actions and industry, and call us benefactors of dental science and lovers of our profession; and future generations of scientific operators will cherish our memory, and call us worthy of the honorable title of D. D. S.

